# An affinity-directed protein missile system for targeted proteolysis

**DOI:** 10.1098/rsob.160255

**Published:** 2016-10-26

**Authors:** Luke J. Fulcher, Thomas Macartney, Polyxeni Bozatzi, Annika Hornberger, Alejandro Rojas-Fernandez, Gopal P. Sapkota

**Affiliations:** 1 MRC Protein Phosphorylation and Ubiquitylation Unit, School of Life Sciences, University of Dundee, Dow Street, Dundee DD1 5EH, UK; 2 Center for Interdisciplinary Studies on the Nervous System and Institute of Medicine, Universidad Austral de Chile, Valdivia, Chile

**Keywords:** ubiqutination, proteolysis, nanobody, VPS34, VHL

## Abstract

The von Hippel–Lindau (VHL) protein serves to recruit the hypoxia-inducible factor alpha (HIF1α) protein under normoxia to the CUL2 E3 ubiquitin ligase for its ubiquitylation and degradation through the proteasome. In this report, we modify VHL to engineer an affinity-directed protein missile (AdPROM) system to direct specific endogenous target proteins for proteolysis in mammalian cells. The proteolytic AdPROM construct harbours a cameloid anti-green fluorescence protein (aGFP) nanobody that is fused to VHL for either constitutive or tetracycline-inducible expression. For target proteins, we exploit CRISPR/Cas9 to rapidly generate human kidney HEK293 and U2OS osteosarcoma homozygous knock-in cells harbouring GFP tags at the VPS34 (vacuolar protein sorting 34) and protein associated with SMAD1 (PAWS1, aka FAM83G) loci, respectively. Using these cells, we demonstrate that the expression of the VHL-aGFP AdPROM system results in near-complete degradation of the endogenous GFP-VPS34 and PAWS1-GFP proteins through the proteasome. Additionally, we show that Tet-inducible destruction of GFP-VPS34 results in the degradation of its associated partner, UVRAG, and reduction in levels of cellular phosphatidylinositol 3-phosphate.

## 1. Introduction

The degradation of many proteins by the ubiquitin-proteasome system (UPS) plays a fundamental role in protein turnover and the maintenance of cellular homeostasis [1,2]. The system uses the sequential action of E1-ubiquitin-activating enzyme, E2 ubiquitin-conjugating enzymes and E3-ubiquitin ligases to attach ubiquitin chains onto target proteins and mark them for degradation by the proteasome [3,4]. The human genome encodes two E1 enzymes, around 50 E2 enzymes and over 600 E3 ubiquitin ligases [5–7]. The substrate specificity of the UPS is determined by the substrate-recognition elements within the E3 ubiquitin ligase complexes [8,9]. The Cullin RING (really interesting new gene) E3 ubiquitin ligases (CRLs) constitute the largest family of E3 ubiquitin ligases that contribute to the UPS [8,10,11].

The CRL family constitutes seven evolutionarily conserved members, termed CUL1, CUL2, CUL3, CUL4A, CUL4B, CUL5 and CUL7, which share parallel structural and catalytic features [8,12]. The CULs are bound in a complex by selective adaptor and substrate-receptor subunits as well as one RING E3 ligase protein, RBX1/2 [8,11,12]. All CRLs are activated by the covalent attachment of a ubiquitin-like modifier termed NEDD8 through Neddylation, which requires its cognate E1, E2 and E3 enzymes analogous to the ubiquitylation process [13]. A selective inhibitor of the NEDD8-activating enzyme (E1), MLN4924, inhibits the activation of CRLs in cells [10]. The nature and number of substrate receptor subunits for each CRL complex define the range of targets they ubiquitylate for proteasomal degradation in cells. Upon binding the cognate substrate receptor subunit, the CRL substrate is positioned in proximity to the RBX1/2 RING E3 ligase and the respective E2-Ub conjugates, which can efficiently ubiquitylate the substrate, marking it for rapid degradation by the proteasome [8,12,14]. For CUL1 and CUL7, SKP1 serves as an adaptor, whereas one of numerous F-BOX domain proteins functions as a substrate receptor [12]. The F-BOX domain associates with SKP1 in the CUL1/7-complex. Similarly, CUL2 exploits proteins Elongin B/C as adaptors, and VHL serves as the substrate receptor. Under normoxic conditions, VHL binds to hydroxy-proline modified HIF1α and brings HIF1α in close proximity to RBX1 for its ubiquitylation [15,16]. The VHL-BOX domain of VHL, incorporating residues T152-H191 of human VHL, mediates its association with the CUL2/Elongin B/C complex [15]. CUL5 functions in an analogous way to CUL2 except that it exploits SOCS proteins as substrate receptors [15].

Molecular studies on CRLs have established proximity-driven ubiquitylation of substrates by RBX E3 ubiquitin ligases as the key mode of action for their degradation [8,17]. This has allowed the exploitation of the CRL-mediated UPS for the development of novel therapeutics, such as proteolysis targeting chimeric molecules (PROTACs), which employ selective small molecule chimeras that serve to recruit a target protein (e.g. a protein kinase) to a substrate receptor of the CRL system (e.g. VHL) to facilitate the CRL E3 ubiquitin ligase to ubiquitylate the target protein, subsequently marking it for degradation [18,19]. The successful application of some PROTACs [19,20] demonstrates that the CRL system can be exploited for degradation of target proteins as long as they can be recruited selectively to the substrate receptors of the CRL system.

The advancement of the CRISPR/Cas9 genome editing methodologies has made it relatively easy to achieve gene knockouts in somatic cells [21]. However, one key limitation of the CRISPR/Cas9 genome editing technology is that it is impossible to knock out genes that are essential for cell survival or proliferation. It is estimated that there are over 1800 genes for which achieving knockouts with conventional CRISPR/Cas9 methodology is not possible [22]. CRISPR/Cas9 can be used to efficiently knock in GFP tags to both alleles of essential genes provided the proximity/presence of the tag does not adversely affect the target expression or protein function. The fluorescence allows for rapid isolation of fluorescent-positive cells through fluorescence-activated cell sorting (FACS) [23]. Such fluorescent-tagged proteins driven by endogenous promoters not only facilitate robust localization, proteomic and biochemical studies on the target proteins, but also form the basis for their targeted degradation in this study.

The past few years have seen a dramatic rise in the development and use of small (approx. 15 kDa), single chain polypeptide antibodies derived from camelid species (often called nanobodies) against different antigens [24–26]. Many high-affinity and selective polypeptide nanobodies have been generated against fluorescent proteins, including GFP, mCherry and DSred, which selectively bind their targets with low nanomolar affinities [24,25,27]. A key advantage of these small polypeptide nanobodies is that they can be packaged into cDNA plasmids for expression in multiple cell systems and they retain their specificity and affinity for their targets. In this study, we have employed CRISPR/Cas9 to generate cell lines in which we have knocked in a GFP-tag on endogenous VPS34 and PAWS1/FAM83G genes. In these cells, by using the anti-GFP nanobody attached to VHL, we direct the endogenously GFP-tagged VPS34 and PAWS1 to ubiquitin-mediated destruction through the CRL machinery. VPS34 is the only known class III phosphatidylinositol 3-kinase that generates phosphatidylinositol 3-phosphate (PI3P) on endosomal membranes. It is essential for cell survival and plays a key role in membrane trafficking, autophagy and intracellular signalling [28,29]. PAWS1 is a poorly characterized protein that modulates BMP signalling and transcription [30]. By combining the CRISPR/Cas9 genome editing technology and the CRL ubiquitin-proteasome system, we demonstrate, in this study, that it is possible to direct selective target proteins to near-complete degradation. We also demonstrate that the system represents a simple and robust means of investigating the functional consequences of the loss of target proteins and their associated complexes. We name this approach the affinity-directed protein missile (AdPROM) system.

## 2. Results

### 2.1. The affinity-directed protein missile system is effective for degradation of selective endogenous proteins

**Figure 1.**
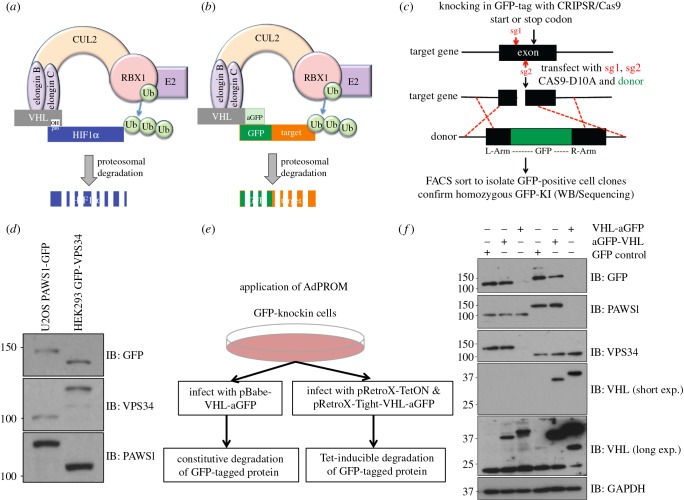
The affinity-directed protein missile (AdPROM) system degrades target proteins in different cell lines. (*a*) Schematic describes how the CUL2-E3 ligase complex results in ubiquitylation and degradation of its native target, hydroxy-proline modified HIF1α, under normoxic conditions. (*b*) Schematic of the exploitation of the CUL2 E3 ligase machinery for AdPROM using anti-GFP nanobody (aGFP) to degrade GFP-tagged proteins. (*c*) Strategy for rapidly knocking in a GFP tag on target proteins in somatic cells using CRISPR/Cas9. (*d*) Western blot analysis of extracts from PAWS1-GFP and GFP-VPS34 knockin U2OS and HEK293 cells, respectively, using the indicated antibodies. (*e*) Schematic shows the application of AdPROM using pBABED-Puro (for constitutive expression) or pRetroX-TetON (for Tet-inducible expression) retroviral infection systems to introduce the VHL-aGFP in GFP-knockin cells. (*f*) A proof-of-principle demonstration of the efficacy of the AdPROM system in the degradation of GFP-VPS34 and PAWS1-GFP from knockin HEK293 and U2OS cells, respectively. Cells infected with control retroviruses (GFP) or retroviruses encoding aGFP-VHL or VHL-aGFP were lysed. Extracts (20 µg protein) were subjected to resolution by SDS–PAGE and transferred to PVDF membranes, which were analysed by western blotting with the indicated antibodies.

Under normal oxygen conditions, the CUL2 E3-ligase complex constitutively ubiquitylates and degrades the protein HIF1α, which is hydroxylated on proline residues by prolyl hydroxylase enzymes for its recognition by VHL [8,16,31] (figure 1*a*). The proximity of VHL-bound hydroxylated HIF1α to the RING-E3 ligase RBX1 in the CUL2 machinery is sufficient for its ubiquitylation by RBX1 [8]. We engineered the AdPROM construct so that VHL was modified by adding an anti-GFP (aGFP) nanobody [26] to either the N- or the C-terminus of VHL and cloned it in to a mammalian expression pBABED retroviral vector. In principle, upon expression of these constructs in cells, the expression of the anti-GFP nanobody tethered to VHL would be predicted to recruit any GFP-tagged protein to the CUL2 E3 ligase machinery (figure 1*b*). Provided the GFP-tagged protein expressed in cells is then suitably positioned for RBX1-mediated ubiquitylation, the AdPROM system would be predicted to cause its ubiquitin-mediated proteasomal degradation (figure 1*b*).

In order to generate cell lines that harbour an endogenous GFP-tag on the target protein, we have optimized protocols for rapidly knocking in GFP tags onto the endogenous gene loci, using CRISPR/Cas9. Using a simple targeting strategy (figure 1*c*), we can efficiently yield homozygous GFP knockins at either the N- or C-terminus of any gene in somatic cells in around three weeks. We accomplished homozygous knockins of GFP tags at the N-terminus of VPS34 in HEK293 cells, and the C-terminus of PAWS1/FAM83G in U2OS cells, as demonstrated by immunoblotting respective extracts with both anti-GFP antibody or antibodies recognizing endogenous VPS34 and PAWS1 proteins (figure 1*d*). Addition of a GFP tag causes disappearance of protein detection at the predicted, native molecular weight, whereas detection is confirmed at a higher molecular weight accounting for the additional GFP tag (figure 1*d*).

Next, we introduced the AdPROM system (figure 1*b*) in the GFP-VPS34 and PAWS1-GFP cells (figure 1*d*), via retroviral infections using a control vector, or vectors encoding the aGFP-VHL or VHL-aGFP polypeptides positioned downstream of a constitutive promoter (figure 1*e*). The expression of endogenous GFP-VPS34 and PAWS1-GFP was then monitored in cell extracts by immunoblotting with either anti-GFP antibody or antibodies recognizing VPS34 and PAWS1 (figure 1*f*). While the control vector and the vector encoding aGFP-VHL did not result in loss of GFP-VPS34 and PAWS1-GFP protein levels, in cells expressing VHL-aGFP, the levels of GFP-VPS34 and PAWS1-GFP proteins were depleted to near undetectable levels (figure 1*f*). Furthermore, in HEK293 cells in which endogenous PAWS1 is not modified by a GFP knockin, and similarly in U2OS cells in which endogenous VPS34 is unmodified, the expression of VHL-aGFP did not change the levels of native PAWS1 and VPS34, respectively, suggesting that VHL-aGFP only targets GFP-tagged proteins for destruction (figure 1*f*). Interestingly, the positioning of the aGFP to the C-terminus of VHL but not the N-terminus appears to be crucial for orientating the GFP-VPS34 and PAWS1-GFP for the RBX1 E3 ubiquitin ligase machinery to ubiquitylate these proteins (figure 1*f*). Consequently, we used the VHL-aGFP polypeptide as the effective orientation for the AdPROM system for subsequent experiments.

In order to confirm that overexpression of VHL and aGFP alone did not affect endogenously GFP-tagged proteins, we performed immunoblotting on PAWS1-GFP U2OS cells, which were infected with vectors harbouring GFP control, VHL alone control, aGFP alone control or VHL-aGFP. As expected, only VHL-aGFP expression resulted in robust PAWS1-GFP degradation, whereas neither VHL nor aGFP on their own were capable of mediating PAWS1-GFP destruction (electronic supplementary material, figure S1). As aGFP alone can still bind to the GFP-tagged proteins in cells, potentially compromising their function in other non-degradative ways, we have used GFP as a control for most of our studies. To demonstrate that the substrate recruitment for AdPROM was mediated through aGFP and increase the versatility of the system, we replaced aGFP with an independently derived, distinct cameloid anti-GFP nanobody, aGFP16 (electronic supplementary material, figure 2*a*) [24]. The two anti-GFP nanobodies recognize distinct structural elements within the GFP beta-barrel structure [24,26]. The AdPROM system harbouring VHL-aGFP16 when expressed in cells also resulted in efficient degradation of GFP-VPS34 and PAWS1-GFP, whereas aGFP16 alone and VHL alone did not (electronic supplementary material, figures S2*b*,*c*). Furthermore, aGFP16-tethered sepharose beads efficiently pulled down endogenous GFP-VPS34 and PAWS1-GFP, but not untagged proteins, from cell extracts and in the process completely depleted them from the respective flow-through extracts (electronic supplementary material, figure S3).

### 2.2. AdPROM exploits the CRL ubiquitin-proteasome pathway for target protein degradation

**Figure 2.**
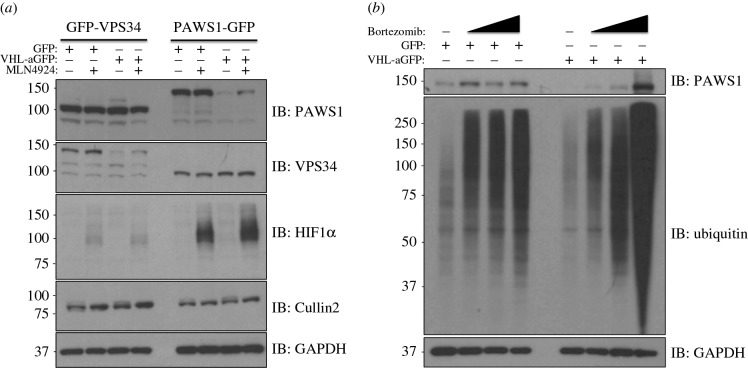
Inhibition of Cullin Neddylation and the proteasome rescues protein degradation through AdPROM: (*a*) retroviral infections of HEK293 and U2OS cells, harbouring GFP-VPS34 and PAWS1-GFP knockins, respectively, were performed to allow the expression of either the control (GFP) or VHL-aGFP polypeptide. Infected cells were then treated with either DMSO or the pan-Cullin Neddylation inhibitor MLN4924 (1 µM) for 6 h, as indicated. Extracts (20 µg protein) were resolved by SDS–PAGE, transferred to PVDF membranes and subjected to western blotting for PAWS1, VPS34, HIF1α and Cullin2 expression as shown. GAPDH was included as a loading control. (*b*) Control (GFP) or VHL-aGFP PAWS1-GFP cells described in (*a*) were treated with 10 µM Bortezomib for 0, 4, 10 or 24 h prior to lysis. Extracts (20 µg protein) were resolved by SDS–PAGE, transferred to PVDF membranes and subjected to western blotting using antibodies against PAWS1, total ubiquitin and GAPDH control as indicated.

In order to determine whether the loss in levels of the GFP-VPS34 and PAWS1-GFP by VHL-aGFP was indeed mediated by the CUL2-CRL machinery, we treated the cells with the pan-Cullin Neddylation inhibitor MLN4924. As expected, treatment of both HEK293 and U2OS cells with MLN4924 resulted in robust inhibition of CUL2 Neddylation and stabilization of its endogenous target, HIF1α (figure 2*a*). Under these conditions, the loss in levels of GFP-VPS34 and PAWS1-GFP was partially rescued by treatment of cells with MLN4924, whereas, as expected, no changes were observed in control cells (figure 2*a*). Importantly, the expression of VHL-aGFP had no effect on the levels of endogenous HIF1α, suggesting the AdPROM system does not interfere with the endogenous CUL2 E3 ubiquitin ligase machinery (figure 2*a*). To verify that the AdPROM-mediated degradation of PAWS1-GFP was mediated by the proteasome, control cells or cells expressing VHL-aGFP were treated with the proteasome inhibitor Bortezomib for 4, 10 and 24 h prior to lysis. PAWS1-GFP and total ubiquitin levels were stabilized in a time-dependent manner in cells expressing VHL-aGFP, whereas no significant change was observed in control cells (figure 2*b*). Interestingly, enhanced levels of PAWS1-GFP smears and total ubiquitin were observed in VHL-aGFP expressing cells but not control cells after 24 h of Bortezomib treatment (figure 2*b*), suggesting accumulation of ubiquitylated PAWS1 upon inhibition of the proteasome.

### 2.3. AdPROM can be adapted for inducible degradation of target proteins

**Figure 3.**
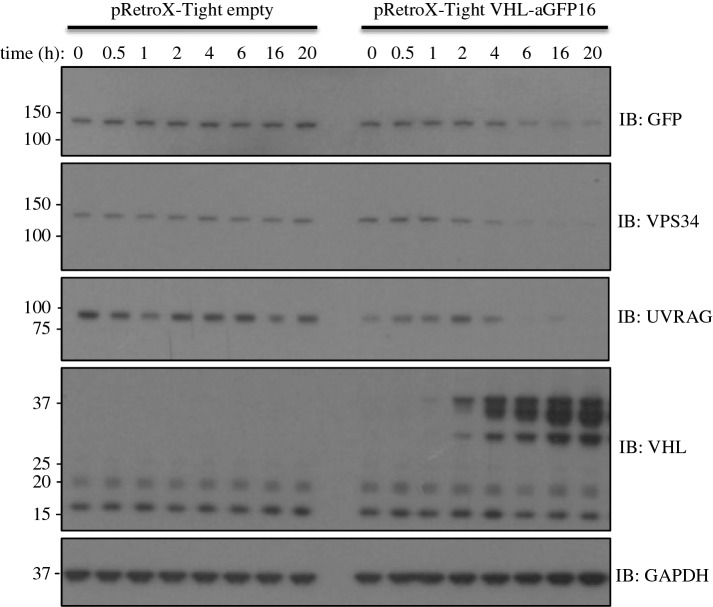
Adapting AdPROM for tetracycline-inducible degradation of target proteins: GFP-VPS34 knockin HEK293 cells were first infected with the pRetroX-Tet-ON advanced vector (Clontech) and selected for the expression of Tet-transactivator. Cells were then infected with either pRetroX-Tight empty vector control or pRetroX-Tight vector encoding VHL-aGFP16. Cells were then treated with 2 µg ml^−1^ doxycycline for the indicated time points prior to lysis. Extracts (20 µg protein) were resolved by SDS–PAGE, transferred to PVDF membranes and subjected to western blotting, using antibodies against GFP, VPS34, UVRAG and VHL, as indicated. Anti-GAPDH antibody was included as a loading control.

Having demonstrated the efficacy of the AdPROM system for constitutive degradation of target proteins, we sought to develop an inducible AdPROM system. We employed the pRetroX Tet-ON retroviral tetracycline-inducible vector system to clone VHL-aGFP16. Using this system, the GFP-VPS34 knockin HEK293 cells infected with either control Tet-ON empty vector or Tet-ON vector carrying VHL-aGFP16 both displayed similar levels of GFP-VPS34 and no detectable levels of VHL-aGFP16 in the absence of doxycycline (figure 3). In control cells, as expected, a time course of doxycycline treatment over 20 h did not alter the levels of endogenous GFP-VPS34, its associated partner UVRAG, VHL or GAPDH proteins. In Tet-ON VHL-aGFP16 infected cells, doxycycline treatment induced the expression of VHL-aGFP16 in a time-dependent manner, with detectable levels observed at 2 h (figure 3). The increased levels of VHL-aGFP16 expression correlated with reduction in levels of GFP-VPS34 protein in a time-dependent manner, with reduced GFP-VPS34 levels relative to untreated controls detected at 2 h after doxycycline treatment and sustained at the lowest levels after 6 h (figure 3). Excitingly, the expression of endogenous UVRAG, a regulatory component of the VPS34 kinase complex, mirrored the degradation of GFP-VPS34, suggesting that an inducible AdPROM has the potential to destroy protein complexes when individual components are targeted (figure 3). The levels of GAPDH employed as controls were unaffected by doxycycline-induced expression of VHL-aGFP16 (figure 3). These observations suggest that the AdPROM system can be adapted to achieve inducible degradation of target proteins, which could be part of pre-assembled complexes, the disruption of which can provide mechanistic insights that a constitutive degradation system might not.

### 2.4. The AdPROM-mediated degradation of GFP-VPS34 inhibits VPS34 function in cells

**Figure 4.**
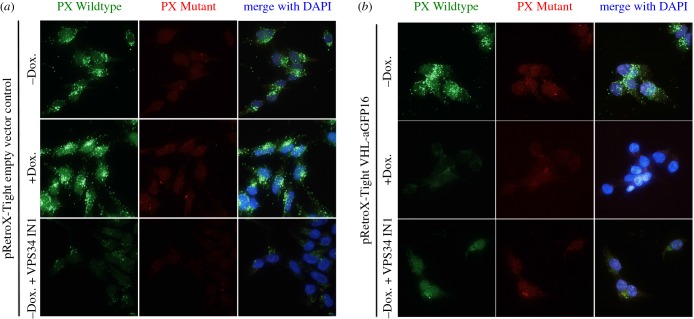
Degradation of VPS34 through AdPROM results in loss of function. (*a*) GFP-VPS34 knockin HEK293 cells expressing the Tet-transactivator were infected with pRetroX-Tight empty vector control. Following selection, cells were seeded onto glass coverslips and either left untreated or treated with doxycycline (2 µg ml^−1^; 24 h) or VPS34-IN1 (2.5 µM, 1 h). Cells were permealized in liquid nitrogen, and fixed in 3.7% (w/v) paraformaldehyde before staining with fluorescently labelled selective PI3P binding (green) and interaction deficient (red) probes as described in the Material and methods section. Samples were mounted on microscopy slides with mounting media containing DAPI (blue). (*b*) As in (*a*), except that the cells were infected with pRetroX-Tight vector encoding VHL-aGFP16. Images were taken using DeltaVision microscopy imaging systems (GE Healthcare) at 60× magnification.

We sought to investigate whether AdPROM-mediated inducible degradation of endogenous GFP-VPS34 affected its cellular function. The catalytic subunit of the class III phosphatidylinositol 3-kinase, VPS34 catalyses the generation of PI3P, which accumulates primarily at the endosomal membranes [32,33]. The endosomal PI3P then serves to recruit proteins containing FYVE and PX domains to mediate downstream signalling. The cellular PI3P can be visualized using fluorescent probes attached to a PX domain. In this study, we used the PX domain of p40phox chemically conjugated to Alexa Fluor 488 [34]. A PI3P-interaction deficient PX domain mutant conjugated to Alexa Fluor 594, employed as negative control, enabled visualization of both wild-type and mutant PX probes in the same samples (figure 4*a*). Using these fluorescent probes, we demonstrate that treating GFP-VPS34 cells infected with Tet-ON control vector with doxycycline for 24 h, which does not degrade GFP-VPS34 (electronic supplementary material, figure S4), did not lead to changes in the PI3P punctate signal compared with untreated controls, whereas cells treated with VPS34-IN1, a selective inhibitor of VPS34, displayed near-complete loss in PI3P puncta (figure 4*a*). Under identical conditions, as expected, no changes were seen with the PX mutant probe (figure 4*a*). In GFP-VPS34 cells infected with VHL-aGFP16 Tet-ON (inducible AdPROM), treatment of doxycycline for 24 h, which results in robust degradation of GFP-VPS34 (electronic supplementary material, figure S4), resulted in near-complete loss in the PI3P punctate signal compared with untreated cells (figure 4*b*). This loss in PI3P puncta was comparable with that observed in cells treated with VPS34-IN1 (figure 4*b*). These observations suggest that AdPROM-induced degradation of GFP-VPS34 impacts the cellular function of VPS34, which is at least comparable with its inhibition using small molecule inhibitors.

## 3. Discussion

In this report, we describe a simple, yet robust methodology for targeted degradation of endogenous proteins in cells. By combining the efficiency of CRIPSR/Cas9 genome editing to rapidly tag endogenous proteins with GFP, and using a single vector in which anti-GFP nanobody is tethered to VHL to target the GFP-tagged proteins for CRL-mediated proteasomal degradation, we provide proof-of-principle evidence that the AdPROM system can target selective proteins and efficiently degrade them in cells, either constitutively or in a Tet-inducible manner.

With the VHL-aGFP AdPROM, we achieved robust degradation of GFP-VPS34 and PAWS1-GFP knockin proteins in HEK293 and U2OS cells, respectively. Interestingly, aGFP-VHL did not lead to degradation of these proteins, suggesting that this orientation is incapable of recruiting the target proteins close to RBX1 in the CUL2-complex for ubiquitylation. We have shown that the degradation of GFP-VPS34 resulted in the loss of its physiological function as judged by reduction in total cellular PI3P levels. Because of the structural similarities between GFP and yellow fluorescent protein (YFP), the anti-GFP nanobodies that we have employed in this study also bind YFP. We have been able to extend the use of VHL-aGFP and VHL-aGFP16 AdPROMs to successfully degrade many other target proteins in cells in which GFP and YFP tags have been knocked in on the target gene loci using CRISPR/Cas9. GFP and YFP knockins are often exploited in cell and animal models as reporters for protein expression, localization and distribution, and to identify interacting partners through proteomic approaches. In order to study the degradation of CUL2 components, including VHL itself, an AdPROM construct harbouring a different CULLIN receptor is recommended.

The proximity-driven ubiquitylation and degradation of target proteins by the CRL ubiquitin-proteasome pathway have also been exploited by PROTACs, which use small, cell-permeable molecules to bring target proteins to the CRL-machinery [18,19]. Because PROTACs can be time consuming and expensive to develop, AdPROMs similar to the one we have described could be exploited to rapidly test the druggability of potential targets as a first step to developing PROTACs. The inhibition of Cullin Neddylation by MLN4924 and the proteasome by Bortezomib could be exploited to manipulate target protein degradation by the AdPROM system that uses the CRL machinery. Isolation and identification of ubiquitylated proteins upon proteasomal inhibition could potentially uncover components of the target protein complex, as targeting VPS34 degradation by AdPROM also resulted in the degradation of its associated partner UVRAG.

One limitation of the AdPROM degradation system in its current form is the length of the affinity probe that can be tethered to VHL without the affinity probe itself being recognized as a substrate by the CUL2 E3 ubiquitin ligase machinery. Because anti-GFP nanobody is tolerated, as observed by its robust expression, it is perhaps safe to assume that a length of around 100 amino acids for the affinity probe is tolerated. More work is being done to understand the minimum determinants of affinity probe tolerance by the system, and to determine if the AdPROM system could be refined to achieve better and more efficient target protein degradation.

While we have focused on target protein degradation in this report, the potential applications of the AdPROM system are vast. In principle, an effective AdPROM system consists of at least two linked elements: an ‘affinity probe’ (such as anti-GFP nanobody in this case) that selectively targets desired proteins; and the ‘payload’, which could constitute a destructive signal (e.g. substrate receptor subunit of any CRL-E3 ligase machinery for target protein degradation), an enzyme (for target protein modification), a subcellular localization signal or any combination of these. The advances in cameloid polypeptide antibody technology will, in the near future, make highly selective nanobodies against specific proteins available that could be exploited as ‘affinity probes’ for AdPROM; as could any protein interaction domain or a signal reader domain. These would bypass the requirement for knocking in fluorescent tags on endogenous proteins. The choice for ‘payload’ could be vast and be applied to address many different research questions on uncovering target protein function and regulation. The simplicity, broad applicability and versatility of the AdPROM system has the potential to make it into an effective tool for investigation of specific endogenous protein isoforms at desired cellular locations, in any cell line.

## 4. Material and methods

### 4.1. Plasmids

A modified Cas9 nickase system [35] was used for the generation of VPS34 and PAWS1-GFP knockins. Optimal sgRNA pairs were identified and chosen on the basis of being as close as possible to the point of GFP insertion while having a low combined off-targeting score (VPS34-sgRNA1: GCTACATCTATAGTTGTGACC (DU52071); sgRNA2: GCCCCATCGCACCGTCTGCAA (DU52082); PAWS1-sgRNA1: GCCTCATCGGATTCTAAACGG (DU48793); sgRNA2: GCCACTGGCTACCGCCCGTCC (DU48826)). Complementary oligos with *Bbs*I compatible overhangs were designed for each and these dsDNA guide inserts ligated into *Bbs*I-digested target vectors; the antisense guides (sgRNA2) were cloned onto the spCas9 D10A expressing pX335 vector (Addgene plasmid no. 42335) and the sense guides (sgRNA1) into the puromycin-selectable pBABED P U6 plasmid (Dundee-modified version of the original Cell Biolabs pBABE plasmid). Donor constructs (VPS34-DU52175 and PAWS1-DU48585) consisting of GFP flanked by approximately 500 bp homology arms were synthesized by GeneArt (Life Technologies); each donor was engineered to contain sufficient silent mutations to prevent recognition and cleavage by Cas9 nuclease. The cDNAs encoding GFP (DU32961), anti-GFP nanobodies (aGFP-DU54218; aGFP16-DU54238) [24,27], human VHL (DU54023), aGFP-VHL (DU54023), VHL-aGFP (DU54221) and VHL-aGFP16 (DU54294) were cloned into pBABED-Puro vectors (Cell Biolabs, modified) for constitutive expression and pRetroX-pTight Tet-ON vectors (Clontech) for tetracycline-inducible expression. The retroviral expression system vectors pCMV-Gag-Pol and pCMV-VSVG constructs were from Clontech. All DNA constructs were verified by DNA sequencing, performed by the DNA Sequencing and Services (MRCPPU, College of Life Sciences, University of Dundee, Scotland, http://www.dnaseq.co.uk) using Applied Biosystems Big-Dye v. 3.1 chemistry on an Applied Biosystems model 3730 automated capillary DNA sequencer. All constructs are available to request from the MRC-PPU reagents webpage (http://mrcppureagents.dundee.ac.uk) and the unique identifier (DU) numbers indicated above provide direct links to the cloning and sequence details.

### 4.2. Antibodies

Antibodies against GFP (S268B), PAWS1 (S876C) [30] and VPS34 (S672B) were generated in sheep and affinity purified by the Division of Signal Transduction Therapy at the University of Dundee. Rat anti-GFP antibody was purchased from Chromotek (cat.: 3H9). Rabbit anti-GAPDH (cat.: 2118) and anti-VHL (cat.: 2738 and 68547) antibodies were purchased from CST. Anti-HIF1α antibody (cat.: 610959) was purchased from BD Transduction Laboratories. Rabbit anti-Ubiquitin antibody (cat.: 106260) was obtained from Dako. CUL2 antibody was from Invitrogen (cat.: 51-1800) and UVRAG antibody was from MBL (cat.: M160-3). For western blot analysis, all primary antibodies were used at 1 : 1000 dilution, except for anti-GFP, anti-Ubiquitin, anti-PAWS1 and anti-GAPDH antibodies, which were used at 1 : 5000 dilution. Horseradish peroxidase (HRP)-coupled secondary antibodies (1 : 5000) were obtained from Santa Cruz.

### 4.3. Cell culture

All cells (HEK293, U2OS and 293-FT) were cultured in Dulbecco's modifified Eagle's medium (DMEM; Gibco) supplemented with 10% fetal bovine serum (Hyclone), 1% penicillin/streptomycin (Lonza) and 2 mM l-glutamine (Lonza), and maintained at 37°C in a humidified incubator at 5% CO_2_. Cells were exposed to compounds or different stimuli as described in the appropriate figure legends. For retroviral production, pBABED and pRetroX retroviral plasmids (6 µg) encoding appropriate proteins were co-transfected with pCMV-gag-pol (4 µg) and pCMV-VSV-G (2 µg) in a 10 cm diameter dish of 70% confluent 293-FT cells. Briefly, plasmids were mixed in 0.6 ml Optimem (Life Technologies) to which 24 μl of 1 mg ml^−1^ polyethylenimine (Polysciences) diluted in 25 mM HEPES pH 7.5 was added. Following 15 s of vortexing and incubation for 15 min at room temperature, the resulting mixture was applied dropwise to the 293-FT cells. The medium was replaced 16 h post-transfection with fresh medium, and retroviruses were collected in the growth medium 24 h later, and filtered using 0.45 µm filters. Target cells (approx. 60% confluent) were infected with the optimized titre of retrovirus medium supplemented with 10 µg ml^−1^ polybrene (Sigma) for 24 h and selected for infection with 2 µg ml^−1^ puromycin thereafter.

For lysis, cells were washed twice in ice-cold phosphate-buffered saline (PBS), scraped on ice in lysis buffer (50 mM Tris–HCl pH 7.5, 0.27 M sucrose, 150 mM NaCl, 1 mM EGTA, 1 mM EDTA, 1 mM sodium orthovanadate, 1 mM sodium β-glycerophosphate, 50 mM sodium fluoride, 5 mM sodium pyrophosphate, 1% (v/v) Triton X-100 and 0.5% Nonidet P-40) supplemented with complete protease inhibitors (one tablet per 25 ml; Roche) and either 0.1% β-mercaptoethanol (Sigma) or 50 mM iodoacetamide (Sigma) to inhibit the DUBs where indicated in the figure legends. Cell extracts were either cleared and processed immediately or snap-frozen in liquid nitrogen and stored at −80°C. The protein concentration was determined in a 96-well format using Bradford protein assay reagent (Pierce).

### 4.4. Generation of GFP-VPS34 and PAWS1-GFP knock-in cells using CRISPR/Cas9 genome editing

HEK293 and U2OS cells were transfected with vectors encoding a pair of guide RNAs (pBABED-Puro-sgRNA1 and pX335-CAS9-D10A-sgRNA2) targeting around either the start codon of VPS34 or the stop codon of PAWS1 (1 µg each) respectively, along with the respective donor plasmids carrying the GFP knockin insert (3 µg). 16 h post-transfection, cells were treated with puromycin (2 µg ml^−1^) for 2 days. The transfection and puromycin treatment process was repeated one more time. Cells were then sorted by flow cytometry and single GFP-positive cell clones were plated on individual wells of two 96-well plates. Viable clones were expanded, and integration of GFP at the target locus was verified by western blotting and genomic sequencing of the targeted locus.

### 4.5. SDS–PAGE and western blotting

Reduced protein extracts (typically 10–20 μg protein unless stated otherwise) or immunoprecipitates (IPs) were separated on 8% SDS–PAGE gels, or 4–12% NuPAGE bis–tris precast gels (Invitrogen) by electrophoresis. Proteins were then transferred onto polyvinylidene fluoride (PVDF) membranes (Millipore). Membranes were blocked in 5% (w/v) non-fat milk (Marvel) in TBS-T (50 mM Tris–HCl pH 7.5, 150 mM NaCl, 0.2% Tween-20) and incubated overnight at 4 °C in 5% BSA-TBS-T or 5% milk-TBS-T with the appropriate primary antibodies. For ubiquitin blots, membranes were denatured in 6 M guanidine hydrochloride (6 M guanidine HCl, 20 mM Tris–HCl pH 7.5) after transfer just prior to blocking [36]. Membranes were then washed in TBS-T and incubated with HRP-conjugated secondary antibodies in 5% milk-TBS-T for 1 h, before a further washing in TBS-T and detection using enhanced chemiluminescence reagent (Thermo Scientific) and exposure on medical X-ray films (Konica Minolta) as described previously [37–39].

### 4.6. PI3P staining using a fluorescent-labelled selective PI3P binding PX domain

For PI3P staining, GST-tagged PX domain (residues 1–148 of p40phox) was expressed in *Escherichia coli* (BL21) and purified over a glutathione column using standard procedures. The recombinant protein was then conjugated to Alexa Fluor 488 as per the manufacturer's protocol. For staining, following doxycycline treatment, GFP-VPS34 knockin HEK293 cells were washed two times with ice-cold PBS and two times with ice-cold glutamate buffer (25 mM HEPES pH 7.4, 25 mM KCl, 2.5 mM Mg-acetate, 5 mM EGTA, 150 mM K-glutamate). Coverslips were snap-frozen in liquid nitrogen and thawed. Coverslips were washed two times more with ice-cold glutamate buffer before fixing with 3.7% (w/v) paraformaldehyde (PFA) in 200 mM HEPES pH 7.4 for 30 min at RT. PFA was quenched by two washes and one 10 min incubation in DMEM containing 10 mM HEPES pH 7.4. Samples were washed twice in blocking buffer (1% (w/v) BSA in PBS) before being incubated in blocking buffer for 15 min. Coverslips were incubated for 1 h at RT with 5 µg ml^−1^ PX domain-Alexa Fluor-488 conjugate (diluted in blocking buffer) and washed three times in blocking buffer. Coverslips were washed once more in ddH_2_O prior to mounting with ProLong Gold antifade mountant. Selectivity was conferred through counter-staining with a PI3P interaction deficient mutant PX domain probe chemically conjugated to Alexa Fluor 594 [34]. Images were captured using DeltaVision Imaging Systems (GE Healthcare) at 60× magnification.

## Supplementary Material

Supplementary Text and Figures for An Affinity-directed PROtein Missile (AdPROM) system for targeted proteolysis
