# Supplementary material for: An affinity-directed protein missile system for targeted proteolysis
Source: Open Biol. 2016 Oct 26;6(10):160255. doi: 10.1098/rsob.160255 (PMC5090066; doi:10.1098/rsob.160255)
Supplement: Supplementary Text and Figures for An Affinity-directed PROtein Missile (AdPROM) system for targeted proteolysis [file rsob160255supp1.pdf]

An Affinity-directed PROtein Missile (AdPROM) system for targeted proteolysis

Luke J. Fulcher[1], Thomas Macartney[1], Polyxeni Bozatzi[1], Annika Hornberger[1], Alejandro Rojas-Fernandez[2] and Gopal P. Sapkota[1]*

[1]MRC Protein Phosphorylation and Ubiquitylation Unit, School of Life Sciences, University of Dundee, Dow Street, Dundee DD1 5EH, UK.

[2]Center for Interdisciplinary Studies on the Nervous System & Institute of Medicine, Universidad Austral de Chile, Valdivia, Chile.

* Address correspondence to: g.sapkota@dundee.ac.uk

Supplementary Figures and Figure Legends

Supplementary Figure 1: Only VHL-aGFP, but not GFP, aGFP or VHL alone controls, induces the degradation of GFP-tagged proteins: PAWS1-GFP knockin U2OS cells infected with control retroviruses encoding GFP, aGFP, or VHL only, or AdPROM retroviruses encoding aGFP-VHL or VHL-aGFP were lysed. Extracts (20 µg protein) were subjected to resolution by SDS-PAGE and transferred to PVDF membranes, which were analyzed by Western blotting with the indicated antibodies.

Supplementary Figure 2: A distinct anti-GFP nanobody tethered AdPROM (VHL-aGFP16) also causes the degradation of GFP-tagged proteins: GFP-VPS34 HEK293 and PAWS1-GFP U2OS cells were infected with retroviruses encoding GFP, aGFP16 alone, VHL alone, or VHL-aGFP16 were lysed. Extracts (20 µg protein) were subjected to

resolution by SDS-PAGE and transferred to PVDF membranes, which were analyzed by Western blotting with the indicated antibodies.

Supplementary Figure 3: aGFP16 IPs completely deplete GFP-tagged proteins from extracts: GFP-VPS34 HEK293 and PAWS1-GFP U2OS cells were lysed and cleared extracts subjected to immunoprecipitation using aGFP16-coupled sepharose beads. The input and post-IP flow-through extracts (20 µg protein) were subjected to resolution by SDS-PAGE and transferred to PVDF membranes, which were subsequently analyzed by Western blotting with the indicated antibodies.

Supplementary Figure 4: Tet-inducible AdPROM degrades GFP-VPS34 upon doxycycline treatment: GFP-VPS34 knockin HEK293 cells expressing the Tet-transactivator infected with either the pRetroX-Tight empty vector or the pRetroX-Tight vector encoding VHL-aGFP16 vector were treated with 2µg/ml doxycycline for the indicated time points prior to lysis. Extracts (20 µg protein) were resolved by SDS-PAGE transferred to PVDF membranes, and subjected to Western blotting using antibodies against GFP, VPS34, and VHL as indicated. Anti-GAPDH antibody was included as a loading control.

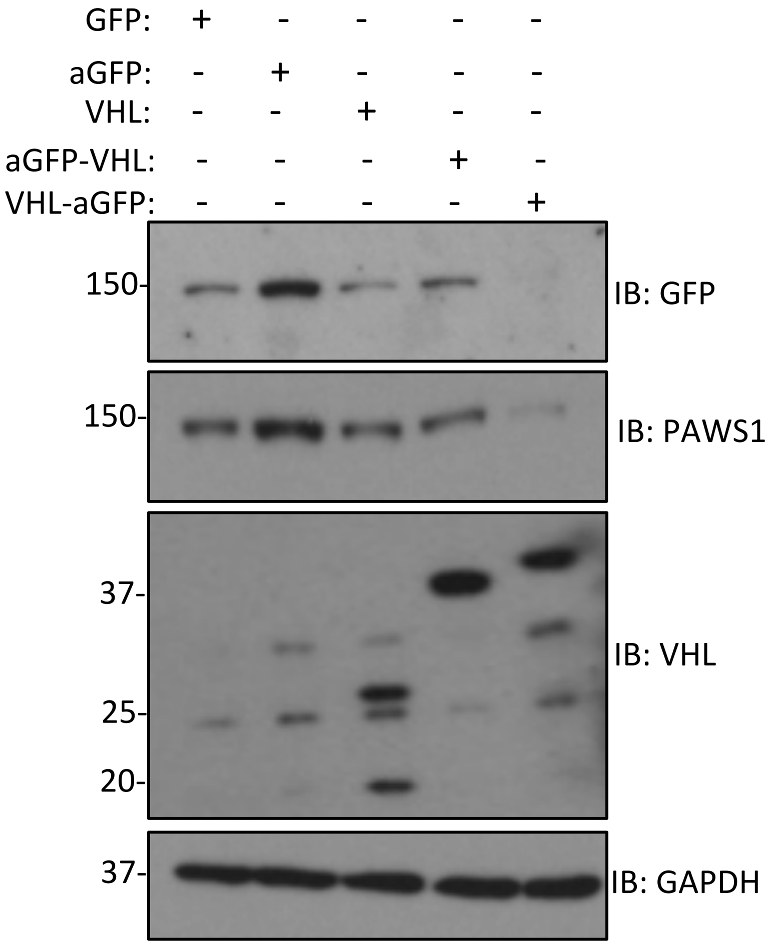

Fulcher et al Supplementary Figure 1

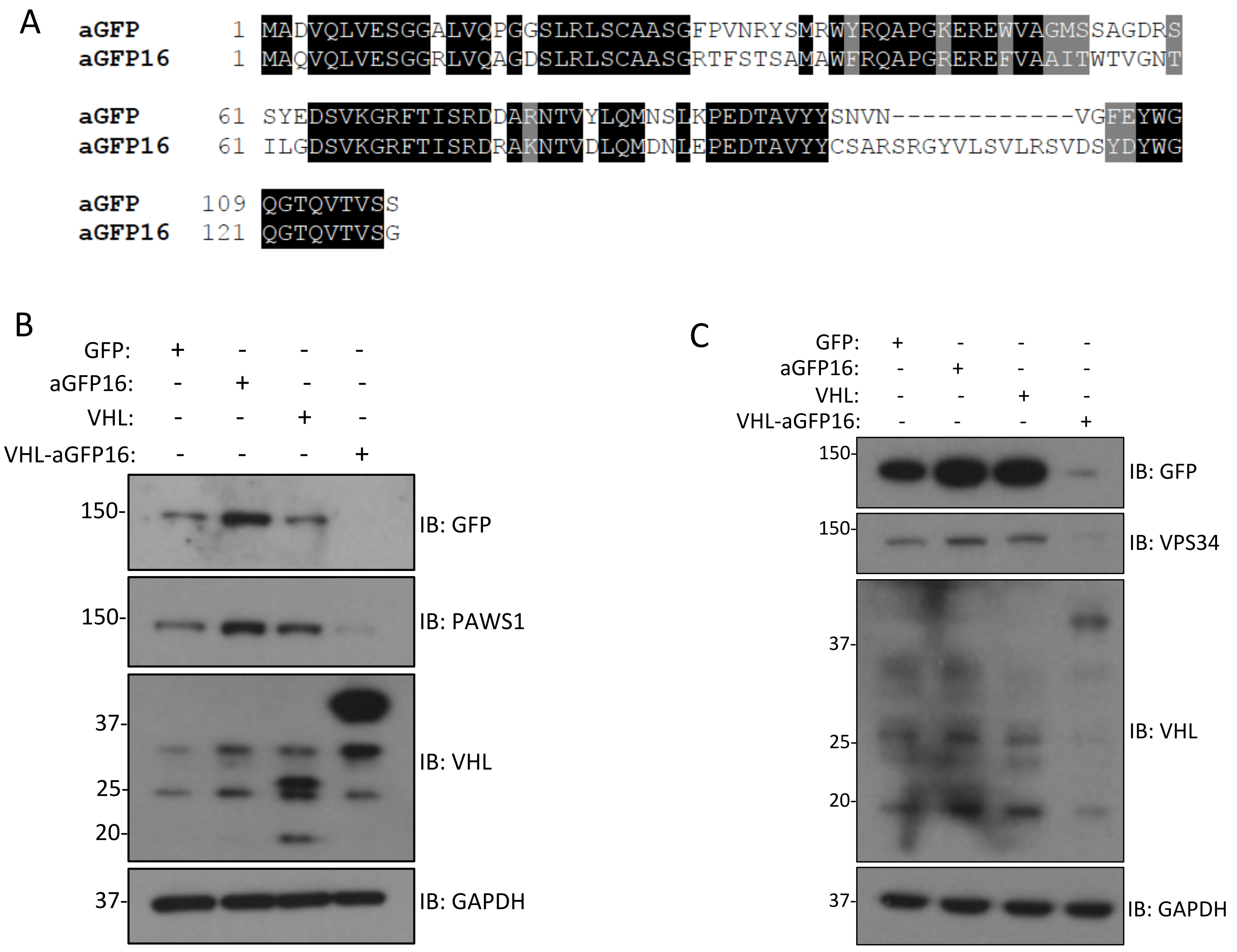

Fulcher et al Supplementary Figure 2

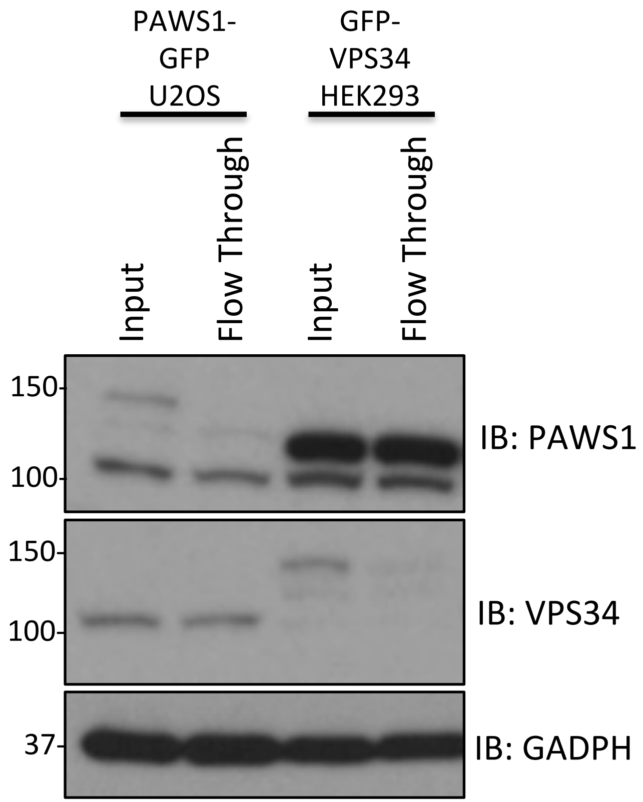

Fulcher et al Supplementary Figure 3

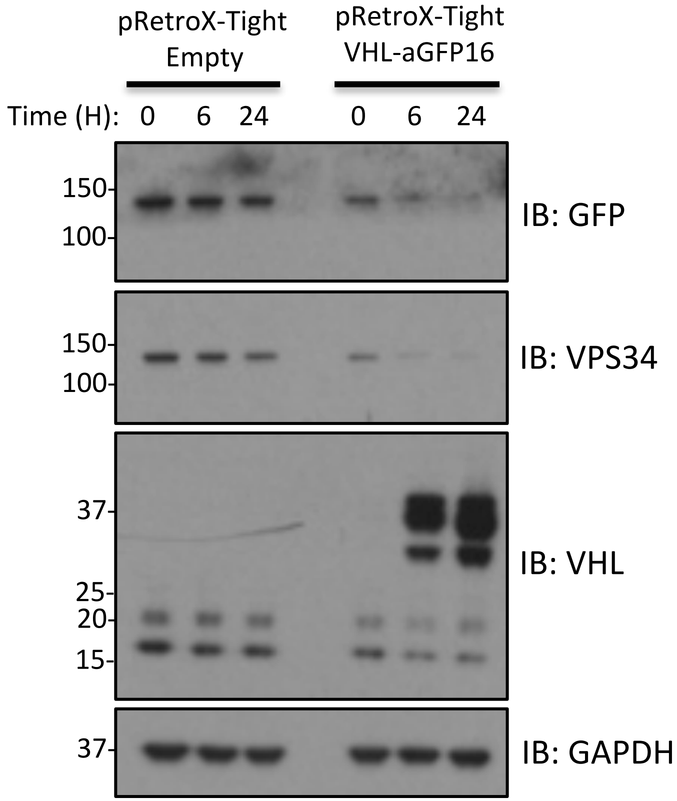

Fulcher et al Supplementary Figure 4
